# Diseases in free-ranging bats from Germany

**DOI:** 10.1186/1746-6148-7-61

**Published:** 2011-10-18

**Authors:** Kristin Mühldorfer, Stephanie Speck, Gudrun Wibbelt

**Affiliations:** 1 Leibniz Institute for Zoo and Wildlife Research, Research Group of Wildlife Diseases, Berlin, Germany; 2 Bundeswehr Institute of Microbiology, Munich, Germany

## Abstract

**Background:**

The emergence of important viral diseases and their potential threat to humans has increased the interest in bats as potential reservoir species. Whereas the majority of studies determined the occurrence of specific zoonotic agents in chiropteran species, little is known about actual bat pathogens and impacts of disease on bat mortality. Combined pathological and microbiological investigations in free-ranging bats are sparse and often limited by small sample sizes. In the present study about 500 deceased bats of 19 European species (family *Vespertilionidae*) were subjected to a post-mortem examination followed by histo-pathological and bacteriological investigations. The bat carcasses originated from different geographical regions in Germany and were collected by bat researchers and bat rehabilitation centers.

**Results:**

Pathological examination revealed inflammatory lesions in more than half of the investigated bats. Lung was the predominantly affected organ (40%) irrespective of bat species, sex and age. To a lesser extent non-inflammatory organ tissue changes were observed. Comparative analysis of histo-pathology and bacteriology results identified 22 different bacterial species that were clearly associated with pathological lesions. Besides disease-related mortality, traumatic injuries represented an additional major cause of death. Here, attacks by domestic cats accounted for almost a half of these cases.

**Conclusions:**

The present study shows that free-ranging bats not only serve as a reservoir of infectious agents, they are also vulnerable to various infectious diseases. Some of these microbial agents have zoonotic potential, but there is no evidence that European bats would pose a higher health hazard risk to humans in comparison to other wildlife.

## Background

The growing awareness of Chiroptera as reservoir species for multiple infectious agents [1,2] has increased the interest in bats as vectors of zoonotic pathogens. In contrast to their negative public perception as carriers of highly virulent viruses [3], the critical role of bats in natural and man-made ecosystems is often unrecognized or underestimated [4]. With changing land use many bat species are threatened, and roost and habitat destruction result in increasing human-bat interactions [5].

Although various studies investigated infectious agents and bats, little is known about actual bat pathogens and the influence of diseases on bat mortality. Combined pathological investigations and pathogen detection in free-ranging bats are sparse [6-9] and often limited by small sample sizes. Most reports describe disease incidents in single bats or small numbers of animals caused by parasitic [10-12], bacterial [13,14] or viral infections [15,16]. In Europe, all bat species are registered in the IUCN Red list of endangered species and legislation for nature conservation prohibits invasive sampling of free-ranging individuals for research purposes. The fast decomposition of bat carcasses is another reason why studies on bat diseases are sparse. Pathological changes caused by infectious diseases in bats are rarely visible macroscopically and microscopy is the only way for comprehensive investigation. We established a close cooperation with bat rehabilitation centres where free-ranging moribund animals were immediately stored frozen after their death. The submitted carcasses were well-preserved to allow diagnostic examinations like histo-pathology and bacteriology.

The aim of the present study was the determination of pathological changes and associated pathogens in deceased free-ranging European bat species to evaluate the often assumed potential high health hazard to humans as well as to support veterinarians dealing with injured or sick bats in their clinical investigations. Above all, the data elucidates the importance of diseases in threatened European bat species.

## Methods

### Study site and sample collection

Between 2002 and 2009, 486 deceased free-ranging bats were submitted for post-mortem examination and histo-pathological and bacteriological investigations. The bat carcasses originated from 6 geographical regions in Germany (Berlin greater metropolitan area, Brandenburg, Lower Saxony, Thuringia, Bavaria, and Baden-Wuerttemberg) and were provided by bat researchers and bat rehabilitation centers. Most animals represented individual cases that have been found dead, injured or moribund close to roosting sites or near human habitations. If bats died in care or had to be euthanized for medical reasons, the carcasses were immediately stored at -20°C and transferred to the Leibniz Institute for Zoo and Wildlife Research, Berlin, Germany, for diagnostic investigations [2].

### Pathological investigation

A full necropsy was performed on each bat and all macroscopic findings were recorded. For histo-pathological examination, small slices of multiple organ tissues were fixed in buffered 4% formalin, processed using standard methods and embedded in liquid paraffin. Sections were cut at 2-5 μm and stained with hematoxylin-eosin (HE). Further special stains were used depending on microscopic findings.

### Bacteriological investigation

Samples of lung, liver, heart and kidney as well as tissues conspicuous for pathological changes (e.g. enlarged spleen) were plated onto Columbia (5% sheep blood), Chocolate, Gassner, and MacConkey agar (Oxoid, Wesel, Germany) and were incubated at 37°C (Chocolate agar 5% CO_2_) for 24-48 h. Specific culture media and conditions for the isolation of *Yersinia*, *Salmonella *and anaerobic bacteria were used if appropriate. Bacterial isolates were identified using different commercial (Api test systems; bioMérieux, Nürtingen, Germany) and conventional biochemical tests [17] and 16S rDNA gene analysis [18].

## Results

Nineteen different European vespertilionid species (i.e., bats of the family *Vespertilionidae*) comprised the 486 bat carcasses examined (Table 1). The overall sex ratio was 270 males (55.6%) to 179 females (36.8%) with 37 animals in which gender had not been recorded where only formalin-fixed organs were received without gender information. Animals in their first year of life (neonates, juveniles and subadults) represented one third (32.5%, n = 158) of all bat samples. For the determination of the age classes, fully furred bats with milk teeth were classified as juveniles and differentiated from subadult bats by the date of sampling, i.e. June to August, the maternity period, corresponding to juveniles.

**Table 1:** Details on bat samples (n = 486) investigated in this study.

Genus	Species	Number of bats			Sex				Age			
						
		n	%		f	m	**n.d**.		1	2	3	4
*Pipistrellus*	*P. pipistrellus*	138	28.4		46	84	8		1	39	12	86
	*P. nathusii*	33	6.8		8	25	-		-	-	11	22
	*P. kuhlii*	8	1.6		4	4	-		-	2	2	4
	*P. pygmaeus*	4	0.8		1	3	-		-	-	-	4
*Nyctalus*	*N. noctula*	92	18.9		40	48	4		4	5	1	82
	*N. leisleri*	3	0.6		2	1	-		-	1	1	1
*Eptesicus*	*E. serotinus*	53	10.9		13	21	19		1	7	3	42
	*E. nilssonii*	17	3.5		9	7	1		-	2	-	15
*Myotis*	*M. mystacinus*	38	7.8		20	18	-		1	18	12	7
	*M. daubentonii*	20	4.1		11	9	-		-	4	5	11
	*M. nattereri*	17	3.5		4	12	1		-	-	1	16
	*M. myotis*	8	1.6		1	6	1		-	-	-	8
	*M. brandtii*	3	0.6		1	2	-		-	1	1	1
	*M. bechsteinii*	1	0.2		-	1	-		-	1	-	-
	*M. dasycneme*	1	0.2		-	-	1		-	-	-	1
*Plecotus*	*P. auritus*	24	4.9		10	12	2		-	3	5	16
	*P. austriacus*	1	0.2		1	-	-		-	-	1	-
*Vespertilio*	*V. murinus*	23	4.7		7	16			-	3	10	10
*Barbastella*	*B. barbastellus*	2	4.1		1	1	-		-	-	-	2

f, female; m, male; n.d., not determined

1, neonate; 2, juvenile; 3, subadult; 4, adult

### Post-mortem findings

**Figure 1:**
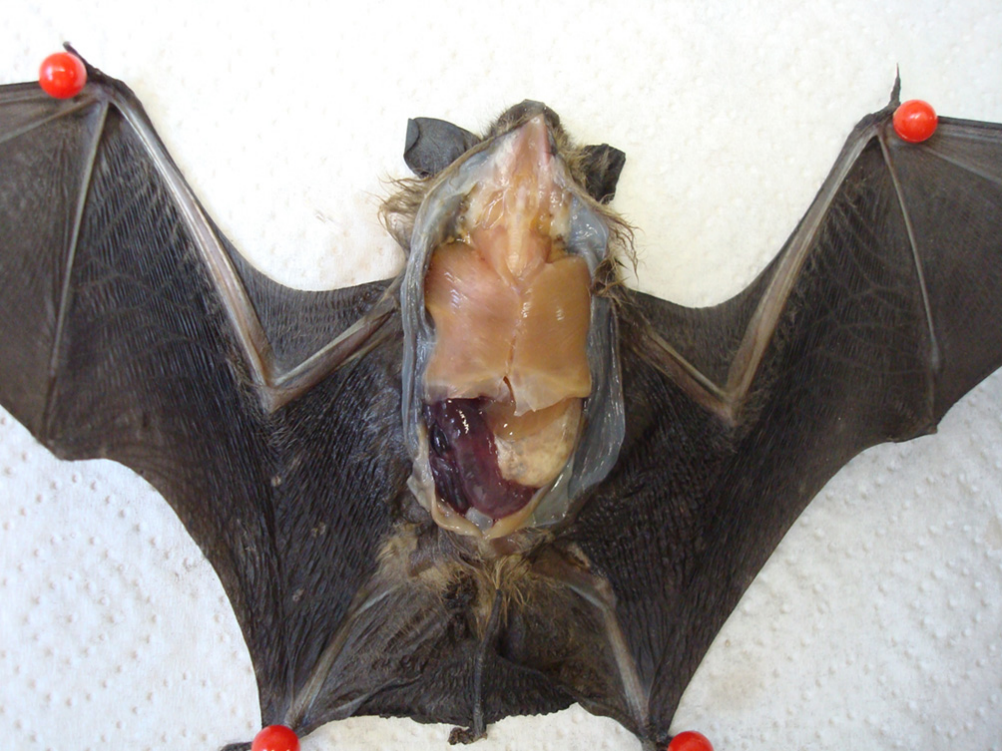
**Photograph of a serotine bat (*Eptesicus serotinus*) showing severe hemorrhagic enteritis and anemia**.

About 39% (n = 189) of all bats investigated in this study revealed mild to severe traumatic injuries, mainly lacerations of the wing membrane (n = 78), open and closed fractures of humerus (n = 31), forearm (n = 50), phalanges (n = 26) and femur (n = 4), loss of extremities (n = 10), rib fractures (n = 5), skull and mandible fractures (n = 4), subcutaneous and intramuscular hematoma (n = 31), and skin abrasions (n = 21). In 24 bats, abdominal or diaphragmatic hernia (n = 14), hemothorax (n = 8), and hemoperitoneum caused by rupture of the spleen (n = 2) were found. Eight bats revealed dislocation of elbow, carpal or knee joints, and in 3 bats, hind leg paralysis was described. In addition to these injuries, enlargement of spleen (n = 66) and/or liver (n = 13) were observed in 16% of bats, and 10 animals revealed moderate to severe hemorrhagic (n = 6) or catarrhal (n = 4) enteritis (Figure 1).

### Histo-pathological findings

**Figure 2:**
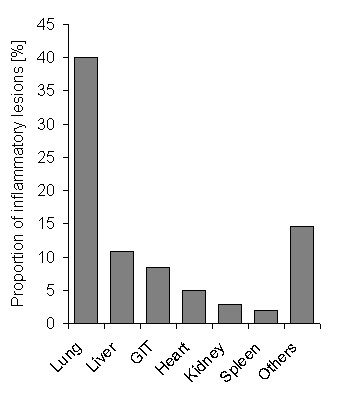
**Proportion of inflammatory lesions in different organs**. Abbreviation: GIT, gastrointestinal tract.

**Figure 3:**
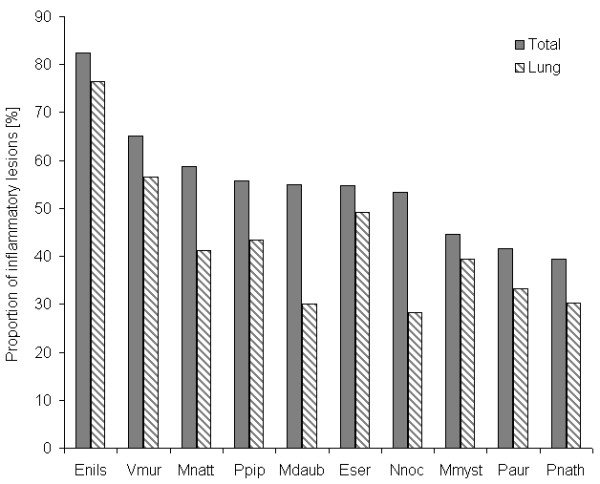
**Proportion of inflammatory changes and lung lesions in different bat species**. Abbreviations: Enils, *Eptesicus nilssonii*; Vmur, *Vespertilio murinus; *Mnatt, *Myotis nattereri*; Ppip, *Pipistrellus pipistrellus*; Mdaub, *Myotis daubentonii*; Eser, *Eptesicus serotinus*; Nnoc, *Nyctalus noctula*; Mmyst, *Myotis mystacinus*; Paur, *Plecotus auritus*; Pnath, *Pipistrellus nathusii*.

Histo-pathological changes were found in almost 84% (n = 408) of free-ranging bats (Additional File 1, Table S1). Among these, inflammatory lesions in one (n = 170) or more (n = 90) organs were observed in 64% of bats, the majority was considered moderate to severe lesions. The lung was the predominantly (40.1%, n = 195) affected internal organ (Figure 2). The prevalence of lung lesions in different bat species ranged from 76.5% (*Eptesicus nilssonii*, 4/17) to 28.3% (*Nyctalus noctula*, 26/92) (Figure 3). An overview of all main histo-pathological findings and causes of death grouped by the different bat species is listed in table 2.

**Table 2:** Major causes of death, histo-pathology and etiology findings in European bat species.

Bat species	Cause of death	(%)	Main histo-pathological findings	Bacterial infection	(%)
Common pipistrelle (*Pipistrellus pipistrellus*)	Trauma^†^	(42.0)	1. Mild to severe interstitial pneumonia	*Pasteurella multocida *	(68.2)
	Organ lesions^‡^	(23.9)	2. Mild to severe enteritis	*Enterococcus faecalis*	
	Bacterial infection	(9.4)	3. Mild to severe multiorgan parasitosis	*Enterobacteriaceae*	
	Others*	(24.6)		*Bacillus cereus*	
Nathusius' pipistrelle (*Pipistrellus nathusii*)	Trauma^†^	(36.4)	1. Mild to severe interstitial pneumonia	*Pasteurella multocida*	(28.6)
	Organ lesions^‡^	(18.2)	2. Necroses (liver, kidney)	*Staphylococcus aureus*	(28.6)
	Bacterial infection	(18.2)	3. Mild to severe multiorgan parasitosis	*Enterobacteriaceae*	
	Others*	(27.2)			
Common noctule (*Nyctalus noctula*)	Trauma^§^	(70.7)	1. Mild to moderate interstitial pneumonia	*Enterobacteriaceae *(*Salmonella *species, *Escherichia coli*)	
	Organ lesions^‡^	(12.0)	2. Mild to severe multiorgan parasitosis	*Staphylococcus aureus*	
	Bacterial infection	(4.3)	3. Mild to severe enteritis		
	Others*	(13.0)	4. Foreign body granulomatous glossitis^*f*^		
			5. Pulmonary arterial hypertension		
Serotine bat (*Eptesicus serotinus*)	Trauma	(22.6)	1. Mild to severe interstitial pneumonia	*Enterococcus *species	(55.6)
	Organ lesions	(20.8)	2. Inflammatory cell infiltrates (liver)	*Enterobacteriaceae*	
	Bacterial infection	(13.2)	3. Mild to severe multiorgan parasitosis	*Pasteurella multocida*	
	Others*	(43.4)	4. Mild to severe enteritis	*Aerococcus viridans*	
Northern bat (*Eptesicus nilssonii*)	Organ lesions	(41.2)	1. Mild to moderate interstitial pneumonia	*Enterococcus faecium*	
	Trauma	(11.8)	2. Mild to severe enteritis		
	Bacterial infection	(5.9)	3. Inflammatory cell infiltrates (liver)		
	Others*	(41.1)			
Parti-coloured bat (*Vespertilio murinus*)	Organ lesions	(30.4)	1. Mild to moderate interstitial pneumonia	*Pasteurella multocida*	(42.9)
	Bacterial infection	(26.1)	2. Necroses (various organs)	*Enterococcus *species	(42.9)
	Trauma^†^	(21.7)	3. Fibrinous suppurative peri-/epicarditis	*Enterobacter *species	
	Others*	(21.7)	4. Severe multiorgan hemosiderosis		
					
Brown long-eared bat (*Plecotus auritus*)	Bacterial infection	(20.8)	1. Mild to severe interstitial pneumonia	*Pasteurella multocida*	(40.0)
	Organ lesions^‡^	(16.7)	2. Necroses (various organs)	*Enterobacteriaceae*	
	Trauma^†^	(12.5)			
	Others*	(50.0)			
Whiskered bat (*Myotis mystacinus*)	Trauma^†^	(26.3)	1. Mild to severe interstitial pneumonia	*Pasteurella multocida *	(33.3)
	Bacterial infection	(15.8)	2. Renal/intestinal coccidiosis	*Enterobacteriaceae*	
	Organ lesions^‡^	(5.3)	3. Mild bile duct proliferation	*Enterococcus faecalis*	
	Others*	(52.6)		Clostridium sordellii	
Daubenton's bat (*Myotis daubentonii*)	Trauma	(50.0)	1. Mild to moderate interstitial pneumonia	*Cedecea davisae*	
	Organ lesions^‡^	(10.0)	2. Mild to focal severe enteritis		
	Others*	(40.0)	3. Severe necrotizing hepatitis		
Natterer's bat (*Myotis nattereri*)	Trauma	(35.3)	1. Mild interstitial pneumonia	*Enterococcus faecalis*	
	Organ lesions^‡^	(11.8)	2. Mild to moderate enteritis		
	Bacterial infection	(5.9)			
	Others*	(47.0)			

^† ^Mainly due to cat predation.

^‡ ^Pathological changes of unknown etiology.

^§ ^Mainly due to roost destruction, trapped individuals and wing fractures of unknown cause.

* Pulmonary edema, anemia, dehydration, hypoglycemia, hypo-/hyperthermia, no significant findings.

^*f *^Associated with chitinous body parts of prey insects [30].

#### Respiratory and cardiac lesions

**Figure 4:**
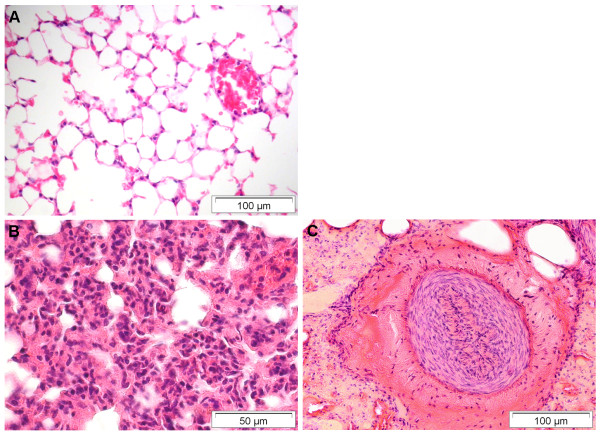
**Pulmonary lesions found in deceased bats from Germany**. A) For comparison: Normal lung tissue with thin alveolar septa. B) Severe interstitial pneumonia due to *Pasteurella multocida *infection characterized by mixed neutrophilic and mononuclear infiltration of alveolar walls. C) Pulmonary arterial hypertension with marked thickening of the artery's media. HE stain.

In the lung, the main inflammatory lesion was mild to severe interstitial pneumonia in almost 38% (n = 182) of bats, predominantly characterized by mixed neutrophilic and mononuclear infiltration of alveolar septa (87.4%, n = 159) (Figure 4b). At least 23% (n = 41) of interstitial pneumonia cases were associated with bacterial infection by members of the families *Pasteurellaceae*, *Enterobacteriaceae *and *Streptococcaceae*. Five animals revealed granulomatous pulmonary lesions caused by larval nematodes (n = 3), secondary bacterial infection due to *Bacillus cereus *(n = 1), or inhaled foreign bodies (plant material) (n = 1). Non-inflammatory lesions were seen in 5 other bats, i.e. mild to severe pulmonary arterial hypertension (n = 4) (Figure 4c) and anthracosis (n = 1). In addition, inflammatory changes (mainly neutrophilic) of the upper respiratory tract were observed in 9 bats. Among these a severe case of purulent rhinitis, sinusitis, and focal ulcerative, suppurative necrotizing glossitis of unknown etiology was seen in a parti-colored bat (*Vespertilio murinus*). The bat was euthanized due to severe dyspnoea.

Main inflammatory changes in the heart were mild to severe peri- and epicarditis (n = 5), mild to severe myocarditis (n = 17) and mild to moderate endocarditis (n = 4) observed in 5% of bats examined. Severe suppurative fibrinous peri- and epicarditis (n = 2), focal suppurative necrotizing myocarditis (n = 1) and suppurative fibrinous effusion of the thoracic cavity (n = 1) were observed as the cause of death in 2 parti-colored bats (*V. murinus*) found independently of each other. In both animals, *Pasteurella multocida *was isolated from heart and thoracic cavity, respectively.

**Figure 5:**
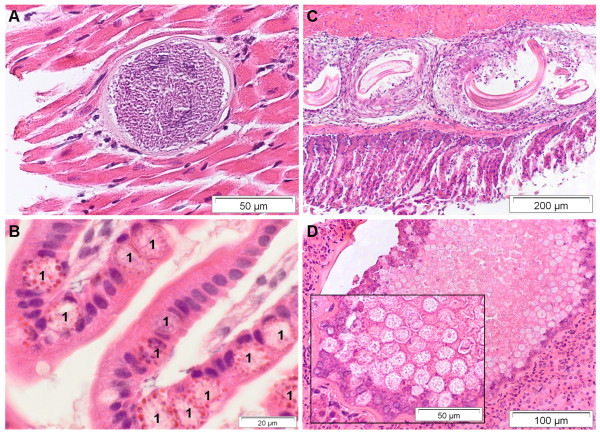
**Endoparasitic lesions within different organ tissues of deceased bats from Germany**. A) Heart. Intramyocardial *Sarcosporidia*-like cyst. B) Duodenum. Intestinal coccidiosis. Coccidia macrogametes and oocysts (1) located within enterocytes. Oil immersion. C) Stomach. Multifocal parasitic granulomas with intralesional nematode larvae. D) Kidney. Renal coccidiosis with cystic tubular dilatation. HE stain.

A cyst-forming *Sarcosporidia*-like protozoan was detected in 5 pipistrelles (*Pipistrellus pipistrellus*, *P. pygmaeus*), 2 noctule bats (*N. noctula*) and a greater mouse-eared bat (*Myotis myotis*). Large cysts containing numerous cystozoites were found in the myocardium (n = 7) (Figure 5a) and the pharyngeal muscles (n = 1).

#### Hepatic, gastro-intestinal and pancreatic lesions

**Figure 6:**
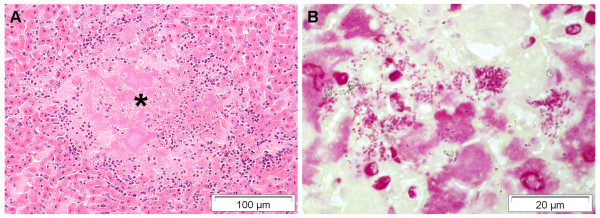
**Severe necrotizing hepatitis associated with *Yersinia pseudotuberculosis *infection in a greater mouse-eared bat (*Myotis myotis*) **[14]. A) Colliquative necrosis (*) demarcated by neutrophils, lymphoid cells and few macrophages. HE stain. B) Numerous intralesional gram-negative coccobacilli. Gram stain. Oil immersion.

In the liver, inflammatory lesions and cell infiltrates were observed in 11% (n = 53) of bats including severe colliquative liver necroses (n = 13), which in 5 animals were clearly associated with bacterial infection (i.e. *P. multocida, Pasteurella *species B, *Yersinia pseudotuberculosis) *(Figure 6). Three bats revealed small granulomatous liver lesions consistent with parasitic larval migration.

Mild to segmentally severe, mainly neutrophilic infiltration of the intestinal mucosa was observed in 7% (n = 34) of bats. Seven animals revealed enlarged villi with marked infiltration of mononuclear cells in the small intestine. In 9 bats, severe trematode infestation or mild to severe intestinal coccidiosis (Figure 5b) were found. Furthermore, focal to multifocal granulomatous gastritis (n = 2) (Figure 5c), serositis (n = 2) and pancreatitis (n = 6) caused by nematode larvae were observed in 2% (n = 10) of bats, while 2 other animals revealed severe granulomatous to abscessating lymphadenitis of unconfirmed bacterial etiology.

#### Splenic lesions

In the spleen, inflammatory lesions were seen in 2% of bats, i.e. mild to severe neutrophilic infiltration (n = 6), severe colliquative necroses (n = 2) that were associated with *P. multocida *or *Y. pseudotuberculosis *infection, respectively, and focal parasitic granuloma (n = 1). Mild to marked activation of the lymphoreticular tissue of the spleen was observed in almost a half (46.9%, n = 228) of bats with obvious follicular hyperplasia in 72% (n = 163). Spleen activation was seen in 55% (n = 144) of bats with inflammatory lesions, and in 55% (n = 103) of injured bats. In 32 animals, moderate to severe follicular hyperplasia was observed without additional significant pathological findings.

#### Kidney and urinary tract lesions

**Figure 7:**
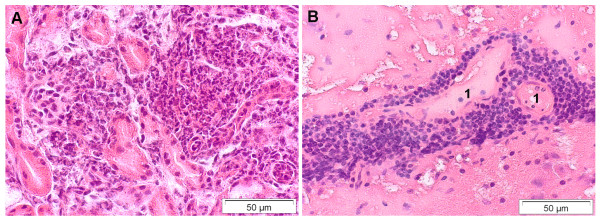
**Suppurative and non-suppurative inflammation of different organ tissues of deceased bats from Germany**. A) Kidney. Severe suppurative necrotizing nephritis due to *Escherichia coli *infection. B) Brain. Rabies encephalitis due to European bat lyssavirus (EBLV-1) infection in a serotine bat (*Eptesicus serotinus*). Cerebrum with marked perivascular non-suppurative inflammation. Blood vessels (1) are surrounded by lymphocytes and plasma cells. HE stain.

In the kidneys, inflammatory changes were noted in 3% (n = 14) of bats. Three animals revealed moderate to severe suppurative necrotizing nephritis that was associated with systemic *P. multocida *(n = 2) or *Escherichia coli *(n = 1) infection (Figure 7a). In another bat, moderate bacterial cystitis and generalized *E. coli *infection were found.

Renal coccidiosis with mild to severe cystic tubular dilatation was observed in 11 bats (2.3%) belonging to 6 different vespertilionid species (*P. pipistrellus*, *P. nathusii, N. noctula, Myotis mystacinus, M. brandtii, Eptesicus serotinus*) (Figure 5d). Additionally, moderate to severe proliferative glomerulopathy (n = 7), moderate proximal tubulonephrosis (n = 1) and severe hydronephrosis (n = 1) were observed in 9 bats.

#### Brain lesions

Moderate perivascular infiltration with mononuclear inflammatory cells was observed in a serotine bat (*E. serotinus*) infected with European bat lyssavirus (EBLV-1) (Figure 7b). Characteristic intracytoplasmic inclusions (Negri bodies) were not evident in that bat. Another bat revealed mild purulent meningitis and encephalitis associated with generalized *Salmonella *Typhimurium infection. Also, multifocal parasitic granulomas caused by nematode larvae were seen in brain and liver samples of a greater mouse-eared bat (*M. myotis*).

#### Tongue lesions

**Figure 8:**
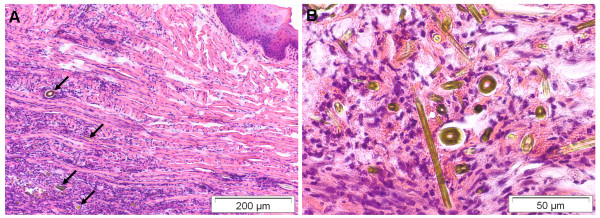
**Severe granulomatous glossitis of a common noctule bat (*Nyctalus noctula*) **[30]. A) Granulomatous inflammation of the tongue's muscle associated with B) intralesional foreign bodies (chitinous insect parts). HE stain.

Inflammatory changes of the tongue were observed in almost 4% of bats (n = 17). Two animals revealed severe necrotizing ulcerative glossitis in the first third of the tongue. Another bat had severe suppurative necrotizing inflammation on the base of the tongue associated with *P. multocida *infection caused by a cat bite. In 7 bats, granulomatous glossitis was caused by intralesional foreign bodies (chitinous body parts of insects as well as bat hairs) (Figure 8). Mild to moderate mononuclear inflammatory cell infiltrates in the salivary glands were seen in 5 bats.

#### Skin, pectoral muscle and skeletal lesions

All investigated skin samples were taken from affected areas of hairy skin. Two animals revealed moderate purulent dermatitis associated with mixed bacterial or bacterial-fungal infection. Further 5 bats had moderate to severe focal ulcerative necrotizing dermatitis. In 11 bats, non-inflammatory orthokeratotic hyperkeratosis of the skin of unknown etiology was observed within areas of sparse hair to alopecia on neck, shoulder, thorax and abdomen.

Histo-pathologic changes of the pectoral muscles were observed in almost 3% (n = 14) of bats and were predominantly characterized by focal or multifocal mixed inflammatory cell infiltration and single muscle fiber degeneration.

Mild to severe arthritis was found in 2 animals with a swollen carpal or knee joint, respectively. Another bat had bilateral protrusions of the mandible of unknown etiology associated with severe osteolysis and abnormal bone formation. Cross section of the mandibular lesions revealed dental fistulas and penetrated chitinous body parts of insects on both sides of the mandible.

#### Metabolic disorders

Single or multiorgan hemosiderosis was observed in 6% (n = 28) of bats. Eight animals revealed mild to severe hepatocellular fatty degeneration (n = 8). In 3 bats, hemolytic anemia was considered as the cause of death because of moderate to severe hemosiderin deposition in liver (Kupffer cells and hepatocytes), spleen (siderocytes) and kidney (tubular epithelium and urinary filtrate).

## Discussion

The present study is the first combined pathology and bacteriology survey performed on deceased free-ranging bats from Germany. Traumatic injuries (39%) and respiratory disease primarily due to pneumonia (40%) were the most frequent lesions detected in 486 bat carcasses examined.

Traumatic injuries have been described as a major cause of death in free-ranging European bat species [6,7,19,20]. In the present study, trauma-associated mortality was observed in almost 40% of deceased bats found in Germany. Fractures and lacerations of the wings were most common in traumatized individuals. About 90% of bone fractures were seen in the upper extremities and almost two thirds were considered compound fractures, reflecting that bat wings are prone to injury due to their large area and delicate structure [21]. Particularly, fractures of humeri and forearms are likely to lead to more severe consequences and subsequently result in death. Additionally, most grounded bats suffer from dehydration and starvation or fall prey to other animals, even if only minor injuries caused them to be grounded.

Domestic cats are frequently involved in trauma cases with cat attacks reported in up to 60% of injured bats [22]. In this study, cat predation was estimated to be responsible for almost a half of traumatic injuries and was often associated with wing lacerations and soft tissue damage followed by wound infection of *P. multocida *[18]. Bat species roosting in buildings (mostly *Pipistrellus *species, *Plecotus auritus*, *V. murinus *and *M. mystacinus*) were primarily affected by cat predation. In contrast, trauma in noctule bats (*N. noctula*) was mainly found in individuals trapped in confined spaces or owing to the destruction of their tree roosts during hibernation by wood loggers.

Pneumonia of varying degrees was the most common histo-pathological finding irrespective of bat species, sex and age. About 90% of interstitial pneumonia in bats was characterized by mixed inflammatory cell infiltrates consistent with an underlying infectious etiology. Almost a quarter of these pulmonary lesions were clearly attributed to bacterial or parasitic infection. However, in most affected lungs neither infectious agents nor viral inclusion bodies were detected during microscopic examination. The lung is a major target organ for numerous infectious agents as well as airborne particles. Bats have an enormous relative lung size and respiratory surface area compared to non-flying mammals [23,24]. The remarkable morphological and functional features of bat lungs are comparable to the avian respiratory system [24], which also seems to be more susceptible to respiratory diseases [25,26]. In both animal taxa, the large respiratory surface and thin blood-gas (tissue) barrier highly increase the respiratory efficiency, but might also predispose bat lungs to injury from environmental toxicants as well as pathogenic microorganisms like in birds [25]. It is discussed whether the length of time bats are kept in captivity without flying might contribute to pulmonary disease due to a lack of exercise [27]. However, in this study the observed high incidence of pneumonia was primarily associated with bats that either were kept only a few days in captivity or had died due to a sudden traumatic event (e.g. wood logging).

The overall prevalence of pulmonary lesions was similar to previous investigations of bats from Austria (38.7%) [11], and the Czech Republic (34.2%) [9], but varied considerably among different species. In our study, noctule bats (*N. noctula*) and Daubenton's bats (*M. daubentonii*) were less affected (28-30%) by pulmonary disease compared to other bat species, while Hajkova and Pikula [9] observed higher prevalence (39%) of pneumonia in *N. noctula*. Most likely such species-specific differences in disease prevalence are caused by a sampling bias, as most bats represented incidental cases and therefore, do not reflect the actual population density of bat species in an investigated area. Nevertheless, variations in roosting ecology and behavior might also influence susceptibility of bats to certain infectious agents [28,29]. Northern bats (*E. nilssonii*) and parti-colored bats (*V. murinus*) appear to be more affected by disease, while trauma-associated mortality was more common in the tree-roosting species *N. noctula *and *M. daubentonii*. Histo-pathological findings (e.g. granulomatous glossitis associated with chitinous parts of prey insects) [30] also seem to be associated with a particular bat species.

In addition to respiratory lesions, various pathological changes were observed in different organs of deceased bats from Germany. Although clinical signs like diarrhea, anemia, skin and urinary tract infections [6,9,20,22,31] and specific histo-pathological changes (i.e. generalized amyloidosis) [32] were reported in individual bats, most inflammatory and non-inflammatory lesions found in this study are newly described in vespertilionids. Furthermore, common bacterial diseases like *E. coli*-urinary tract infection, systemic salmonellosis or yersiniosis [14] associated with characteristic pathological lesions are also first mentioned in free-ranging bats.

Parasitic nematodes infesting different organs and the bloodstream have already been described in bats [7,11,31,32]. For example Daffner [7] identified a nematode species in the stomach as well as the thoracic and abdominal cavity of a brown long-eared bat (*P. auritus*). Another study reported severe cardiopulmonary filariosis in 5 adult noctules (*N. noctula*) originating from a bat rescue centre in Vienna, Austria [11]. In our study, disseminated nematode infection with larval migration was noted in 18% of bats infested with endoparasites. Cross sections of nematode larvae were observed within arteries, capillaries and the right heart atrium as well as in granulomatous lesions of various organ tissues. Bats of 9 different species were affected by larval migration, but large bats like *N. noctula*, *E. serotinus, V. murinus *and *M. myotis *constituted about 80% of all cases.

Renal coccidiosis has sporadically been reported in free-ranging bats [10,12,33]. In European bat species, infection was observed in 4 animals of 4 different vespertilionid species found in Germany [10]. Of the 486 bats examined in the current study, 11 individuals belonging to 6 different species revealed renal coccidiosis. Histo-pathological lesions were consistent with reports from Gruber et al. [10] and Wünschmann et al. [12], while the reported macroscopically visible white foci on the external surface of the kidneys were only seen in 2 animals we examined. Inflammatory cell response was observed in one of these cases and was associated with a cystic cavity filled with amorphous necrotic material. The impact of the renal coccidiosis on bats and its relation to bat mortality remains unclear. Several of such affected bats in this study either had no further significant microscopic or macroscopic indications of disease or had died due to traumatic injuries. It seems likely that a progression of this disease might influence the health status of the infected host, as a half of the bats with renal coccidiosis were in poor body condition.

An additional finding was the prevalence of intramyocardial *Sarcosporidia*-like protozoal cysts observed in 8 bats of 4 different species. A similar protozoan parasite was described once in lesser short-tailed bats (*Mystacina tuberculata*) from New Zealand [8]. This is the first description of an intramuscular *Sarcocystis*-like species in free-ranging bats from Europe.

## Conclusion

The present study provides much new information on diseases in free-ranging bats. Generalized or single organ inflammation was observed in more than 50% of bat carcasses, with at least one third of organ lesions being severe enough for a significant impact on the animals' health. Based on comparative analysis of histo-pathology and bacteriology results, we identified 22 bacterial species that were clearly associated with pathological lesions. At least 11% of all bats presumably died due to bacterial infection. Identifying pathological changes and associated pathogens responsible for morbidity and mortality in free-ranging bats is a first step toward understanding the impact of infectious diseases in threatened European bat species. But more importantly this study provides data indicating that free-ranging bats in Europe can occasionally harbor bacterial pathogens with zoonotic potential. However, we found no evidence to suggest that bats pose a higher disease risk to humans than other wildlife species.

## Competing interests

The authors declare that they have no competing interests.

## Authors' contributions

GW and SS conceived of the study and conducted its design and coordination. KM, SS and GW developed and performed the experiments. GW supervised the pathological analyses. SS supervised the bacteriology analyses. KM analyzed the data and wrote the paper. All authors read and approved the final manuscript.

## Supplementary Material

Additional file 1 **Table S1 Details on pathological changes detected in free-ranging bats from Germany**. Click here for file
